# The Siberian Paleolithic site of Mal'ta: a unique source for the study of childhood archaeology

**DOI:** 10.1017/ehs.2021.5

**Published:** 2021-01-28

**Authors:** Liudmila Lbova

**Affiliations:** Graduate School of International Relations, Peter the Great St Petersburg Polytechnic University, St Petersburg and Novosibirsk State University, Novosibirsk, Russia

**Keywords:** Gender, age, society, anthropomorphic figurines, Upper Paleolithic, Prehistory Art

## Abstract

As a gendered perspective has emerged in wider society over the past 50 or so years, a greater interest in gender- and age-related research in science has similarly occurred, including for the study of the past (archaeology) and the present (ethnology). Here, I focus on the Mal'ta collection – a well-known Ice Age site located in Siberia. In particular, I focus on several mammoth ivory anthropomorphic sculptures which appear to reflect stages of human childhood, including infancy and the teenage years. These sculptures feature realistic elements, including proportions of each phase of childhood consistent with anthropometric data, details of clothing and accessories, and special benchmarks of puberty. Based on these figurines, I propose a developmental framework of the Paleolithic child from this society. Additionally, I discuss the burial of two children also found at Mal'ta, which provides additional insights into childhood within this Ice Age society. Particular attention is given to artefacts such as the ‘hanging birds’ and animal figurines with a flat base for standing. These artefacts could be interpreted as toys, as amulets for a child's cradle or as family heirlooms, with analogies to such objects preserved in the cultures of the aboriginal population of Siberia and the Far North.

## Graphical abstract

**Figure:**
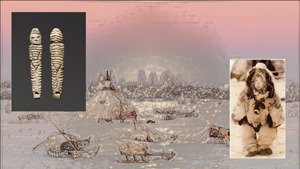

## Introduction

Childhood archaeology is trending in archaeology, cultural anthropology and ethnography. Unfortunately – as elsewhere – investigating past childhoods has not received due attention in Russia, except in bioarchaeology which has studied burials of children in the contexts of large Bronze Age necropolises. In the modern anthropology of childhood, researchers pay attention to the peculiarities of behaviour and relationships with other members of society, the processes of play and creativity, as well as material culture. These aspects have not been extended to Russian archaeological records, however, and in this regard, I would like to express special gratitude to the meeting at Griffith University, Brisbane, Australia (2019) that helped to focus my attention on these aspects in relation to the recovered materials from the Siberian Paleolithic assemblage of Mal'ta.

## Archaeological context

**Figure 1.**
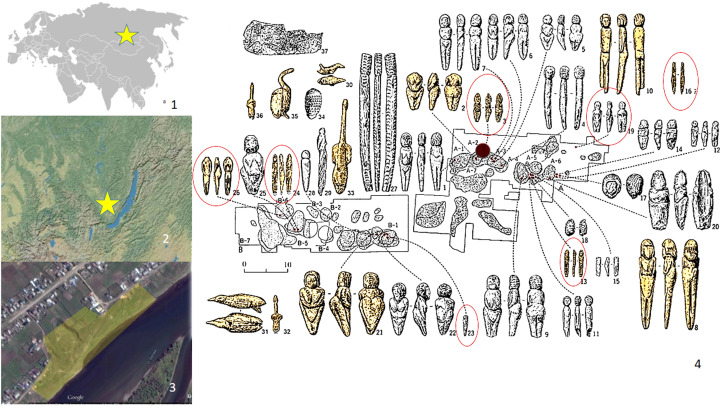
Mal'ta: (1) general location; (2) Baikal region location; (3) modern topographical situation; and (4) plan of M. Gerasimov's excavation. Child figurines are circled in red (N3, 4, 13, 16, 19, 23, 24, 26). The black mark in the centre of the plan shows child burial place (drawing by H. Kato, based on plan by G. Medvedev, 2001).

**Figure 2.**
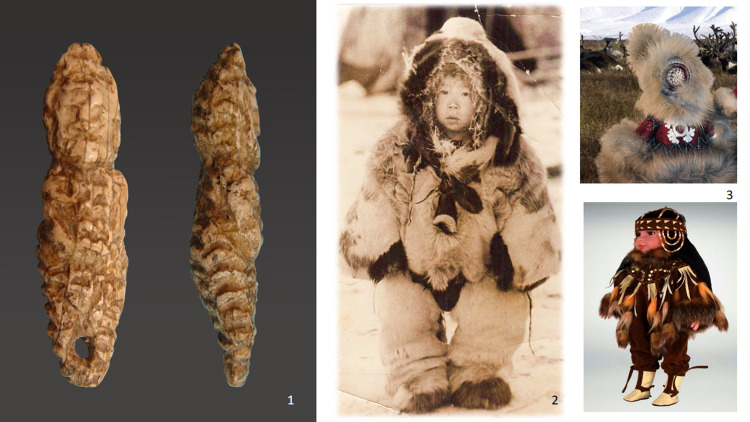
Ivory figurine of a child in fur coverall and ethnographic interpretations. (1) Image of child figurines from the collection of Mal'ta (State Hermitage No370/752); (2) ethnographic photo of the beginning of the twentieth century as an evidence for similar types of clothing; and (3) images of modern Chukchee doll in recent times.

**Figure 3.**
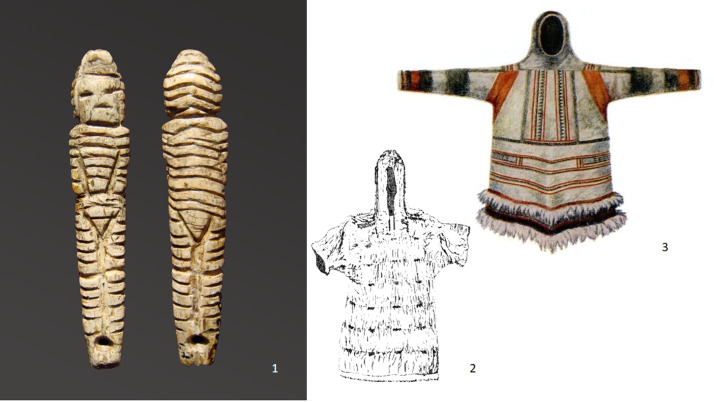
Ivory figurine of a child in coverall and ethnographic interpretations. (1) Images of child figurines from the collection of Mal'ta (State Hermitage No. 370/753); (2) ethnographic evidence for similar types of clothing worn by peoples of this same region in recent times (Bogoraz, 1904, fig. 180); and (3) images of modern Chukchee doll's summer clothes.

**Figure 4.**
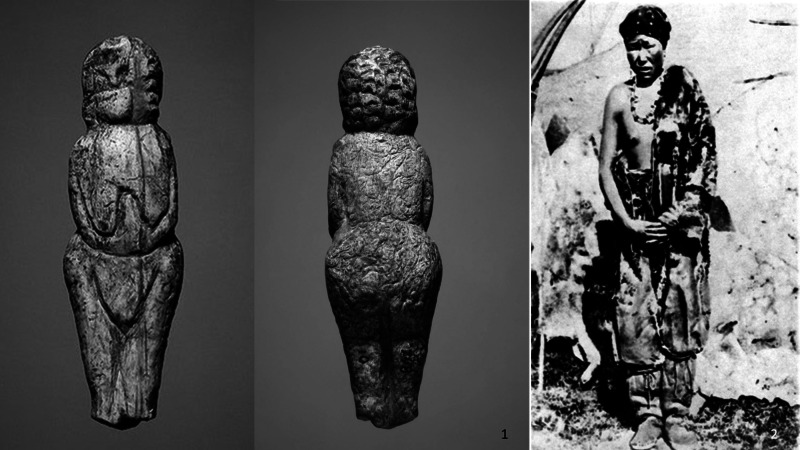
(1) Image of an adult woman from the Mal'ta Collection (Hermitage, No. 370/748); (2) a photograph of northern Indigenous Chukchi woman in summer (Bogoraz, 1904, plate XXVII, fig. 3).

Mal'ta is a multilayered archeological site in the Baikal region of Siberia (Figure 1(1–3)) with cultural deposits ranging from 43,000–41,000 to 12,000 uncal years BP (Lipnina, 2012). The main collection of finds was recovered during the excavations led by M. Gerasimov in 1928–1958 (Gerasimov, 1958), before current excavations, directed by G. I. Medvedev and E. A. Lipnina, focused on dating, microstratigraphy and cultural differentiation of the assemblages. According to the investigations of Gerasimov, the ‘classic’ Mal'ta layer contained a ‘Gravettian-like’ lithic industry with stone and ivory objects recovered from some 15 dwelling structures, all dating from 19,000–23,000 uncal years BP (Gerasimov, 1958; Lipnina, 2012; Kuzmin et al., 2011; Lbova et al., 2017). These finds were found to correspond to layers 8 and 9 – an initial stage of the Last Glacial Maximum owing to recent sections cut during current excavations by G. Medvedev (Lipnina, 2012). This collection contains more than 13,000 artefacts, of which more than 850 items are considered wholly unique evidence for the culture and art of a Paleolithic population. In addition to anthropomorphic figurines, the Mal'ta collection contains over 800 ivory and bone artefacts including different figurines, numerous pendants, objects with ornamental decoration, ivory and stone bracelets, perforated discs, beads and ivory plaques engraved with the representation of a mammoth, and nail-like pins in the same archaeological context (Figs 1–4).

The technocomplexes of the Upper Paleolithic are based on advanced blade technologies with rich, diversified lithic, bone and antler tools predominate. The classic Mal'ta assemblage includes many ‘archaic’ components such as side scrapers, pebble tools and Levallois and discoid cores. As such, the Mal'ta Culture is now regarded as having local roots (Medvedev et al., 1996, Medvedev, 2001; Derevyanko et al., 1998).

During the Upper Paleolithic, specifically during the Ice Age, like in other parts of the Old World, we see evidence for a flourishing culture of reindeer and mammoth hunters, as evidenced by diverse sets of stone blade industries, a rich series of artefacts of bone and antler implements, personal ornaments and mobile art objects. To summarise, Mal'ta is a typica site of Siberian's Ice Age, especially for the middle of the Upper Paleolithic Period.

The Mal'ta collection contains more than 850 items of unique evidence for the culture and art of a Paleolithic population (Table 1). The mammoth ivory anthropomorphic sculptures provide an opportunity to identify several stages of human childhood – from infancy through to teenage-hood. The realistic style of the Mal'ta sculptures characterises the figurines and appears to correlate with anthropometric population data, provide details of clothing and accessories and indicate special benchmarks of puberty within the society. A framework for how a child of this Paleolithic society progressed into adulthood is proposed here based on these artworks.

**Table 1:** Malta-Buret’ collection: artifacts made of ornamental stone, ivory, bone and antler

	Groups	number (ex.)	material	Reference
Evidence of technology for processing organic materials				Medvedev, 2001; Lipnina, 2002, table 5
1.	Blanks	22	ivory	
2.	Shavings, results of cutting	72	ivory	
	Shavings, results of cutting	53	antler	
3.	Flakes	10	ivory, bone, antler	
4.	Blades	13	ivory	
5.	Cut bones	6	polar fox's bone	
	Chipped along the with traces of processing	91	bone	
Finished products and their fragments				Medvedev, 2001; Lipnina, 2002, table 5
1.	Ornamented blades (plaques, discs, tiaras, “buckles”, buttons)	60	ivory	
2.	Bars and points	33	ivory	
	Bars-pins (ornamented)	11*	ivory	
	Bars	20	ivory	
3.	Point, knitting needles, awls	55	ivory	
4.	Needles	70	ivory	
Figurines				Abramova, 1966; Okladnikov, 1968; Medvedev, 2001; Lbova et al., 2017; Lbova, Pankina, 2018; The Paleolithic of Siberia…1998
1.	Anthropomorphic	40*	ivory, antler	
	Adult person	23*	ivory, antler	
	Child and teenager	8*	ivory	
	Heads	4	ivory	
	Blanks	7*	ivory	
2.	Zoomorphic	27*	ivory	
	Bird images	21*	ivory	
	Animal images	3	ivory	
	Fish images	1	ivory	
	Snake images	2	ivory	
Personal ornaments				Medvedev, 2001; Lipnina, 2002, table 5
1.	Pendants	15	teeth, bone, antler	
	Pendants	4	soft stone	
2.	Beads	3	bird's bone	
	Beads	32	fish's bone	
3.	Beads and pendants (necklace)	139	ivory	
4.	Bracelets	10	ivory, stone (torbanite)	
5.	Crystals, pendants, and “buttons”	57	stone (calcite)	
Others				Medvedev, 2001; Lipnina, 2002, table 5
1.	Pebbles (small size)	7*	Stone (nephrite)	
2.	Pieces for painting	6	hematite	
Total		856		

*Malta and Buret sites (together).

## Materials and results

Through combining microscopic analysis (Lbova & Volkov, 2016; Lbova et al., 2017) with modern ethnographic data, it can be proposed that the analysis of these mobile art objects is not only a source of information for the particular material culture of this Paleolithic population of Siberia, but also enlightens the semantic context of mobile art in Mal'ta.

### Anthropomorphic figurines

Thirty-two items in varying degrees of readiness and disposal represent anthropomorphic figurines. The collection contains blanks, objects at the processing stage, objects with some engraved parts and objects ornamented in part or in full. The figurines which are engraved can be broken into the following groups:

1. three-dimensional figures, with shaped body parts – both with and without ornamental elaboration (clothing, accessories);
2. flat figures – with and without ornamentation; and
3. heads – with ornamentation.

Engraved or carved ornamentation is found on the head (*n =* 16), the trunk (*n =* 7), the feet (*n =* 2, although not including pieces that are engraved entirely) or the entire body (*n =* 6) (Lbova, 2017).

Among the figurines of children, all of these subcategories are present, except for isolated heads. The selection of figures that we identified as images of children (infants, adolescents, teenagers) is based on a number of indicators: the overall morphology and proportions of the body, ornamentation and engraving of particular parts of the body, or a special kind of clothing. Analysis of these aspects supports Gerasimov's description of some of the figurines as ‘kinder-garden’ (toddlers, kinder garden-aged children). It should be noted that all the sculptures assigned to the ‘child’ group are ornamented or otherwise engraved to display the main parts of the body (particularly for teenagers) – they are never left blank. In this group, we can confidently identify eight figurines that are definitely young children and adolescents (artefacts are 2–5 cm in size) and two can be assigned as ‘probable teenage images’ (one from Mal'ta and one from Buret’). Young children are determined by the proportions of the body and head, whereas elongated limbs (legs) are seen as typical for identifying adolescents. A characteristic feature for this group is the lack of breasts, which are always present on the adult female figures.

Microscopic analysis allows for identifying the different types of hats, hairstyles, shoes and accessories. These are depicted with thin lines made by a stone burin or other special type of stone knife. The ancient artists used different techniques to highlight the different materials depicted – fur, leather, and special. symbols or decorations. In the realistic elements of clothing and hats, we are apparently seeing details of traditional outerwear of these Ice Age peoples. The most common outerwear depicted on the figurines is fur coveralls – ‘kerkery’, which are still worn by children and women in the extreme north of Siberia (Figure 2(1, 2)). For the Mal'ta figurines, these coveralls are more typical on smaller sculptures (2–5 cm in height). Additionally, all of the figures dressed in coveralls have a disproportionately large head, such proportions being typical for children under five years of age. In other words, these sculptures show small children in clothes that may have been typical for them – coveralls with high hoods. On other sculptures, it is possible to see coveralls made from guts, probably from fish or seals, which people are known in recent times to have worn in the summer in this region (Figure 3).

On two figurines, we can also see bags, and in one case, a traditional backpack with two straps (one over each shoulder). The first figure is probably depicting a teenager, although it does not have much detail so it is not clear if this figure is male or female, yet the proportions of the body suggest that this figure is definitely a teenager. There is no indication of breasts on these pieces, as found on the ‘kinder garden group’.

One of the more interesting details is a figurine which depicts a woman showing one nude breast (Figure 4). This feature was noted in earlier studies where it was read as a symbol of death, or representing an underground realm. On the one hand, this idea is due to the fact that some researchers believe that the Mal'ta figurines are symbol of dead people. However, on the other hand, we can find an ethnographic analogy in the life of the modern Chukchi. As a rule, in summer, nursing women do not cover one breast for constant readiness for feeding their baby (Bogoraz, 1904; Figure 4(2)). As such, we might reinterpret this figurine as a breastfeeding woman.

**Figure 5.**
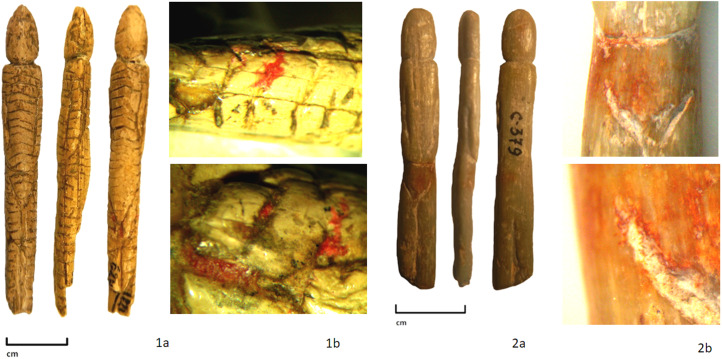
Images of teenage girls: (1a) Mal'ta collection (N 1822/629 State Historical Museum); (2a) Buret collection (С 379, Irkutsk Regional Art Museum); (1b, 2b) macro photos (with magnification of 10×, 20×) of the painting area with ochre.

The use of red paint can be considered a specific technique that marks on the sculpture the transition of children to adulthood, especially girls. One of the artefacts, the figurine of a ‘teenage girl’, appears to be dressed in a one-piece garment with a hood, which covers the entire body and head. This piece is an elongated thin figure. The surface of the statuette is covered with thin horizontal lines and on the front and back there are triangles that imitate the pubis in the front and the tail in the back (this last significantly lower than the pubis). On the face, we see some traces of working with a burin, while other areas are covered with polish. The piece has been decorated with a stone blade (Abramova, 1962; Lbova et al., 2017). The presence of scarlet pigment has been detected using microscopy in the area under the tail, on the right thigh and on the right arm (Figure 5(1a, b)).

Similarly, Buret's image of a young girl with elongated body proportions (characteristic of adolescents) was made from a small pebble of talc. Head, shoulders, body and legs are all depicted. Traces of a chisel are observed, used in forming the contour of the head; a burin was used to engrave the details of the face (left eye) and the contour of the head, as well as to shape the contour of the arms and legs, and the area of the bosom. Diagonal traces of a scraper are found on the back of the head and on the legs in the buttocks area. The maker paid special attention to the bosom zone, which has been carved with great care. Additional emphasis is also found in the form of red colouring applied to the area (Figure 5(2a, b)).

As reported previously (Lbova et al. 2017; Lbova & Volkov 2017), different paints have been identified on the surfaces of the Mal'ta figurines (light rose, pink, red, green, blue and dark blue). Spot of different colours were fixed during excavation, within the structures that retain dwellings and on the surface of the cultural level. A colouristic picture of Mal'ta site presents the widespread use of different natural mineral pigments and artificial paints in different spheres of the live Mal'ta inhabitants, include attention to figurines of pubertal age girls.

### The child burial

**Figure 6.**
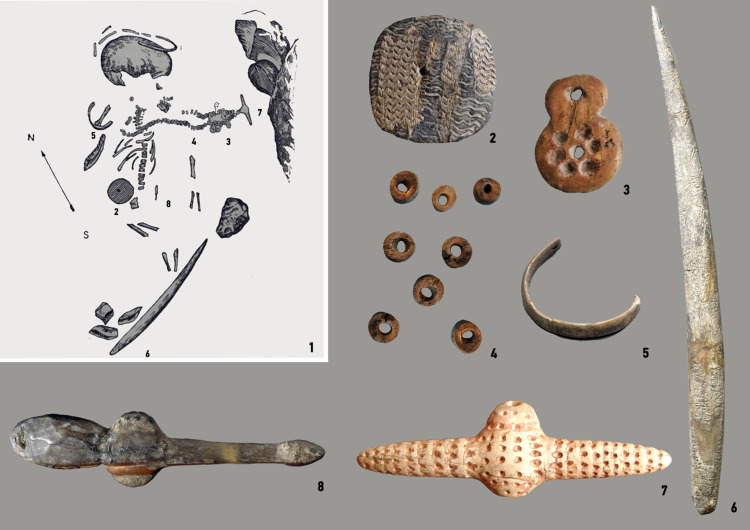
Mal'ta child burial. (1) Material from the Gerasimov excavation (from Gerasimov, 1931). (2–8) Funerary equipment: (2) ornamented disk, (3) 8-image pendant, (4) beads, (5) bracelet, (6) ivory point, (7) central pendant and (8) bird-image pendant (collection of State Hermitage).

The children's burial at Mal'ta is of particular interest when considering evidence for childhood in Siberia's Paleolithic record. The children's skeletons were buried within a slab structure resembling a dug and lined grave (Figure 1). The bottom of the burial was covered with red ochre. Various ornaments and utilitarian objects were found amongst the bones: fragments of a tiara made from mammoth ivory, a rich necklace of 120 bone beads, a large oval-shaped pendant, a round bone plaque with a zigzag pattern and a sculpture of a flying bird. In addition to jewellers, a number of stone tools, as well as a roughly processed flint knife, lay next to the child (Figure 6). Gerasimov was firmly convinced that this inventory of artefacts could not belong to the child himself because of his age, 3–4 years old (Gerasimov, 1931). Owing to his young age, the boy could hardly have created or used them fully. However, there is an additional point of view that such a funerary inventory may well speak of ideas about the afterlife initiation, and the rite of passage into adulthood.

Objects of special meaning, which we consider as ‘prestigious technologies’, accompany the remains of this child. It is worth noting that the child's necklace testifies to various technological and probably culturally bound techniques for raw material processing. The combination of objects within a single complex (burial) suggests a special cultural status of both the deceased himself and the votive artefacts. However, such conclusions were made at a time when children were not considered as an important object of study. Childhood researchers suggest that such things could be considered as toys that children used in life, or objects of special cultural status (Sofaer Derevenski, 2000).

Thanks to paleogenetic studies (Raghavan et al., 2014), it is now known from mtDNA isolated from the child's genetic material that its relationship with Europeans and American Paleo-Indians is at an intermediate position. It is known that in the Mal'tese collection there are other anthropological remains, including the teeth of another child in the burial, which can provide new data on the paleogenetic of this ancient population. The idea that the child can act as a symbol connecting different groups of the population is not excluded. There are some facts that need to be studied and understood. Such a case is known from children's burials in Sungir-site, Russia. Plant DNA analysis materials show a principally different ethnic genesis of each child, who were placed in one grave (Bader 1967; Trinkaus et al., 2015; Sikora et al., 2017; Alekseeva & Bader, 2000).

## Discussion

The artistic realism of Ice Age art presented in the Siberian Mal'ta collection allows for various interpretations of their meaning. The identification of apparently realistic scenes (as compared with modern ethnographic data) in the decorated Paleolithic anthropomorphic sculptures from Mal'ta leads me to the opinion that the chosen style was a way to represent the natural patterns of human life. The choice of attributes reflects specific cultural, environmental and historical conditions behind this particular tradition of material culture (Abramova, 1966, 1995; Delporte, 1979; Lipnina, 2012; Soffer et al., 2000; Filippov, 2004; Lbova et al., 2017).

After more than 150 years of study of Paleolithic portable art, and especially the anthropomorphic figurines, several interpretations can be offered (Marshak, 1991; Barton et al., 1994; Soffer et al., 2000; Art as Behavior, 2014). While some investigators support the idea that they represent ‘magic wishes’ of the owner, or promise of sexual activity, or nostalgia for the departed (deceased) person (Abramova, 1966; Frolov, 1987; Soffer et al., 2000), here, I have supported the idea that the Mal'ta figurines may depict specific living people (live models). All figurines are copies or portraits of particular individuals with characteristic elements of their constitution, clothing, accessories and individual physical type. Personal physiological state (pregnancy) and the age and sex categories of the community (toddlers, children, teenagers, girls, women of reproductive age, old women) support this simple model. These circumstances certainly point to the depiction of a real person, one whom the ancient artist personally knew and chose to portray.

Surprisingly, interpretations of these figurines as reflecting realistic and detailed images of once living persons have been previously lacking in the consideration of these famous pieces. The initial suggestion, that they were a magic or religious item, is more about ritual practice, giving the image magical properties. This idea presents the generalised image of a progenitor (the ancestor's legendary image) as a symbolic expression of blood-related family, as suggested by A. Okladnikov (1968), or as a domestic patron, a spirit in the pantheon of the family (grandma, hostess, mistress of animals) and a universal spirit helper, also for children or progeny (Tokarev, 1961; Frolov, 1987, Cohen, 2003).

In some cases, the figurines appear to represent a tool of astrological operations (Larichev, 1999; Frolov, 1987). However, it is not entirely clear how this idea relates to figurines that are not ornamented, but only have realistic parts of the body or face and elements of clothing.

Gerasimov, who excavated this rare Ice Age settlement, considered a clear connection between anthropomorphic figurines, the habitat and the people who lived in it. All of the figurines were found in the living quarters of the settlement, some even in ritual places within the dwelling, some were covered with a mammoth shovel or sprinkled with ochre (Gerasimov, 1931, 1958). Gerasimov talked a lot about the significance of these unique finds, leaning more towards the idea of those who have gone to another world, creating the memory of the ancestors through sculpture.

Some of the Mal'ta figurines are perforated with a circular hole at the base or an oval slit between the lower legs so that they could be strung and worn upside down as pendants and seen the right way around when held in the hand. However, our microscopic analysis also shows that small holes on the figurines may have had another purpose. We suggest that they must have been firmly attached to clothing so they did not to move, as indicated by the use wear distribution and intensity. The other idea is that they could have been attached to a cradle with leather laces, in keeping with a known tradition among modern Siberian Indigenous groups. Both of these uses were for defense, for guarding the person (or baby) through the use of a protective charm.

It is also worth noting that children could use these objects, alongside other items the remains of which have not been preserved (such as wood, leather, fur, etc.) as toys (Baxter, 2005). Along these lines, a special hypothesis surrounding child's play was formulated for the ‘hanging birds’ and images of animals with a flat base for standing. Similarly, such an interpretation might explain the anthropomorphic figures – which would then be dolls (Filippov, 2004) and could wear made doll-clothing, be painted on the surface or have just the idea of clothing through additional ornaments. Thus, these pieces can be interpreted as toys or amulets for a child's cradle, and analogies for both of these uses are preserved in the cultures of the Indigenous populations of Siberia and the Far North.

Cultural symbols, along with symbols of the body (gestures and language), are all constants of human communication. Study of the Mal'ta anthropomorphic collection in the framework of symbolic interactionism, a concept proposed by Mead (1934), can explain the realistic art style and the ancient artists’ attention to detail (including to the clothing and accessories shown). The cumulative technological and iconographic analysis of these pieces to date leads us to understand that sculptures such as those recovered from Mal'ta – whatever their interpretation – reflect a sustainable element of culture and social communication that determined the artistic style.

## Conclusion

The study of visual techniques used in early Siberian art reveals a number of artistic features, which form a system of developed cultural codes transmitted through symbols and images. One of the main theses of symbolic interactionism is the assertion that the individual. personality is always social, i.e. a person cannot be formed *outside* society. The same idea applies to children. The behaviour of an individual is determined, according to the symbolic concept of communication, by three variables: the structure of the personality, the role of the reference group and the ‘recognition’ symbol. From our current understanding, the Mal'ta figurines are an element of social communication that determines the realism of the artistic style. This realism allows us to talk about the allocation in their mind of the stages of child development (breastfeeding age, children 3–7 years old, adolescents, sexual maturation, adulthood). Therefore, the Mal'ta collection can be considered as a full-fledged archaeological resource for the study of the individual in Childhood Archeology. Most of the collection can be seen on the NSU website http://mal.ta.artemiris.org/.

## Supplementary material

For supplementary material accompanying this paper visit https://doi.org/10.1017/ehs.2021.5. click here to view supplementary material

## Research transparency and reproducibility

Morphology, technology and others characteristics of Mal'ta collection exemplars are present on the website http://malta.artemiris.org/ made by the author and the Laboratory of Multidisciplinary Researching on Prehistory Art of Eurasia's team (Novosibirsk State University).
