# Supplementary material for: The Siberian Paleolithic site of Mal'ta: a unique source for the study of childhood archaeology
Source: Evol Hum Sci. 2021 Jan 28;3:e9. doi: 10.1017/ehs.2021.5 (PMC10427291; doi:10.1017/ehs.2021.5)
Supplement: Supplementary file 1 [file S2513843X21000050sup.zip › S2513843X21000050sup001.docx]

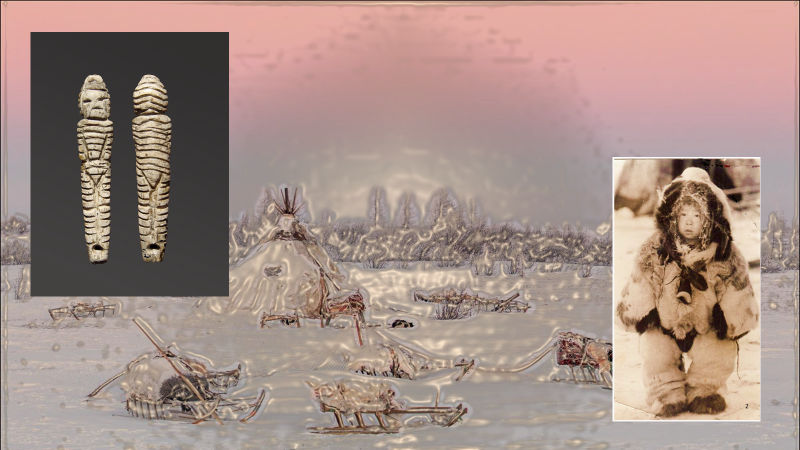


The archeology of childhood is a new and fresh line of research in the field of cultural anthropology, human evolution and cultural development. Our paper presents a unique collection of Mal’ta - a world famous Paleolithic site from Siberia (North-East Eurasia). Archaeological material is represented a rich collection of Prehistoric Art. Sculptures of persons, birds, animals, a lot of decoration items are present as a paleolithic treasure. Among anthropomorphic sculptures, a group of images is highlighted that illustrate children and adolescents. Examination of the figurines with a microscope made it possible to see details of clothing, accessories, hairstyles, and faces. In some cases, micro traces of paint were found on the figurines. In general, realistic artistic images of the Ice Age inhabitants have survived to present day.
