# Supplementary figures and images for: The Siberian Paleolithic site of Mal'ta: a unique source for the study of childhood archaeology
Source: Evol Hum Sci. 2021 Jan 28;3:e9. doi: 10.1017/ehs.2021.5 (PMC10427291; doi:10.1017/ehs.2021.5)

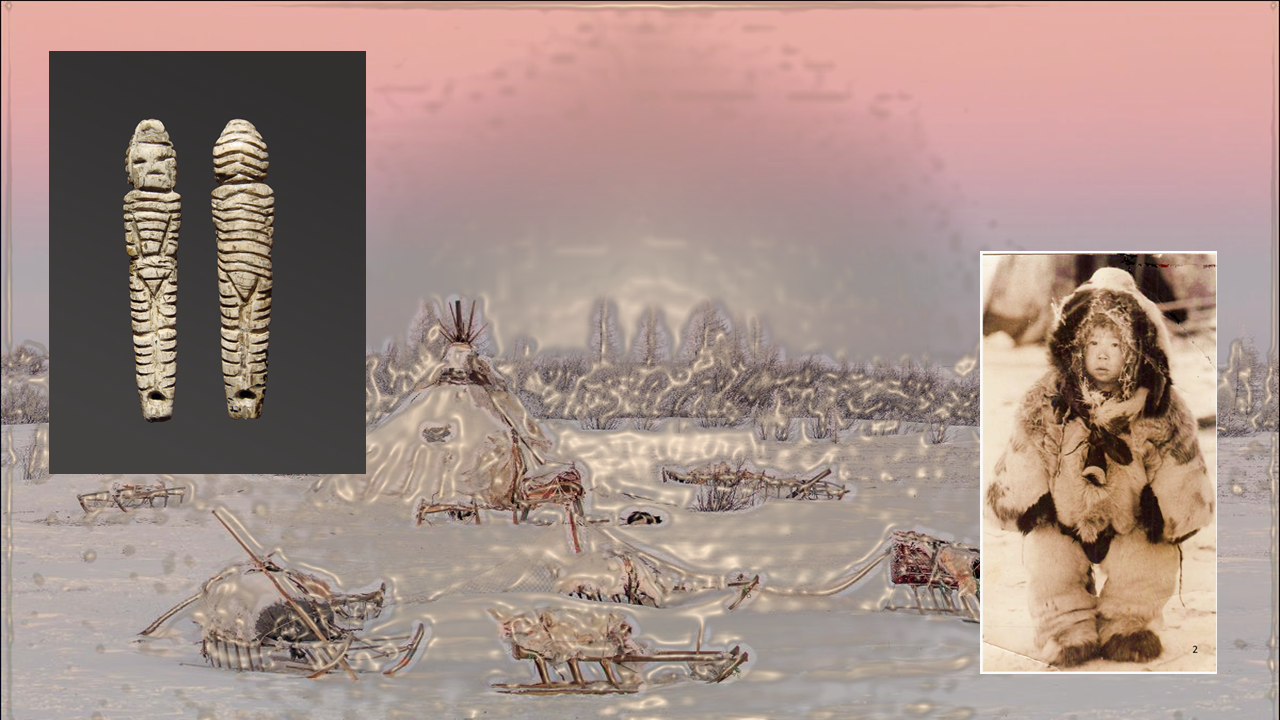

Supplement: Supplementary file 1 [file S2513843X21000050sup.zip › S2513843X21000050sup002.tif]
